# First person – Lucie Perillat

**DOI:** 10.1242/dmm.052050

**Published:** 2025-09-29

## Abstract

First Person is a series of interviews with the first authors of a selection of papers published in Disease Models & Mechanisms, helping researchers promote themselves alongside their papers. Lucie Perillat is co-first author on ‘ Generation and characterization of a mouse model of Becker muscular dystrophy with a deletion of *Dmd* exons 52 to 55’, published in DMM. Lucie is a PhD student in the lab of Dr Ronald Cohn and Dr Evgueni Ivakine at The Hospital for Sick Children (SickKids), Toronto, Ontario, Canada. Her research interests lie in the development of individualized therapies for rare childhood disorders, and the creation of a robust ethical framework to expedite their clinical translation while ensuring the protection of all parties involved – patients and families, scientists, and institutions.


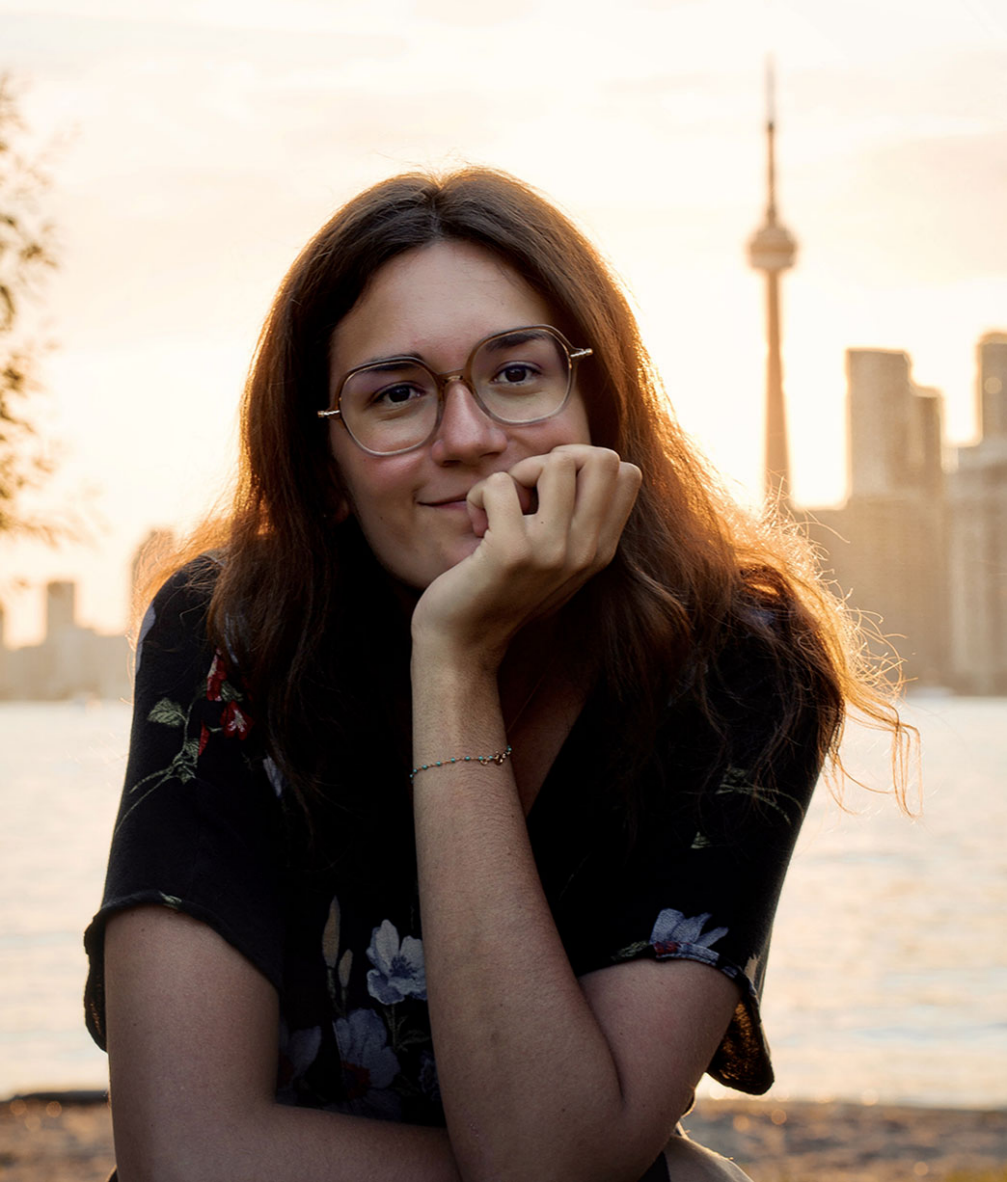


**Lucie Perillat**

**Who or what inspired you to become a scientist?**

My interest in rare diseases research started at the age of 12, after listening to a scientist, Dr Moullier, excitedly describe a breakthrough his team had achieved for patients with Duchenne muscular dystrophy. His intervention was part of a fundraising event for rare diseases research that I had been watching on French television since I was five. Over the years, I had followed the patients and their families, as well as the scientists and clinicians who were trying to develop treatments for these complex disorders. In retrospect, I believe my interest stemmed from a willingness to help patients whose diseases are often neglected and to better understand said diseases, which usually involve several layers of complexity. Although I was convinced that I wanted to become a scientist following Dr Moullier's talk, my parents were more cautious and advised me to make sure a laboratory was the kind of work environment I would thrive in. At the time, they pictured a scientist as someone who works alone, never leaves their bench and always competes with their colleagues. My first encounter with basic research and the laboratory environment made me realize that the work of a scientist is the exact opposite. I remember being inspired by the strong sense of collaboration and teamwork I witnessed during my job-shadowing experience at Atlantic Gene Therapy, a French research unit led by Dr Moullier and focused on making gene therapy treatments a clinical reality. This experience shaped what would become my career ambitions and, retrospectively, was the catalyst for most of my subsequent research experiences.

**What is the main question or challenge in disease biology you are addressing in this paper? How did you go about investigating your question or challenge?**

The main question our paper addresses is to understand the long-term effects of an in-frame Becker muscular dystrophy (BMD)-causing deletion in the *DMD* gene. A major barrier in understanding disease mechanism has been the lack of appropriate BMD animal models. Up until now, there was only one BMD mouse model, named *bmx*, which recapitulates an in-frame deletion of exons 45 to 47 of the *DMD* gene and harbors a typical, mild BMD phenotype, at least at an early age. When it comes to BMD, variants affecting different exons have been shown to be associated with a range of different phenotypes and disease severities. As a result, it is valuable to model and characterize variants across various exons along the *DMD* gene. This led us to investigate another variant located in the second mutational hotspot, specifically the deletion of exons 52 to 55. We used a CRISPR-Cas9 system to generate this new mouse model and characterized it histologically and functionally up until 52 weeks of age. Our results suggest that a truncated dystrophin protein is sufficient to maintain wildtype-like muscle and heart histology and functions in young mice. However, the truncated protein appears insufficient to maintain normal muscle homeostasis and protect against exercise-induced damage at 52 weeks. We performed RNA sequencing pre- and post-exercise to identify several differentially expressed pathways that reflect this abnormal muscle phenotype.

BMD is a rare, neuromuscular disorder that is characterized by progressive loss of muscle functions […] There is no treatment for patients with BMD that stops the disease, only treatments to improve some of the symptoms.

**How would you explain the main findings of your paper to non-scientific family and friends?**

BMD is a rare, neuromuscular disorder that is characterized by progressive loss of muscle functions, including those of the heart, leading to a wide range of clinical presentations, ranging from asymptomatic to requiring a wheelchair and ventilation support. This disease is caused by a variant in a gene called *DMD*, which produces the protein dystrophin. This protein is required to protect muscles from damages induced by movement or exercise. There is no treatment for patients with BMD that stops the disease, only treatments to improve some of the symptoms. A major barrier in understanding the disease and developing treatments has been the lack of BMD animal models. Up until now, there was only one BMD mouse model, named *bmx*, which harbors typical, mild BMD symptoms, at least at an early age. In this paper, we generated an entirely new mouse model of BMD. We analyzed both what the muscles look like and how much activity the mice can do at 12 weeks (young mice) and 52 weeks (old mice). We observed that young mice were not showing any symptoms and had normal looking muscles, meaning that the mutation in our new mouse model is not overly severe. However, at 52 weeks, the mice presented with impaired muscle functions, especially after doing some form of exercise. We looked at muscle cells before and after exercise to try to identify which biological pathways are activated and could explain the impaired muscle functions observed at 52 weeks.

**What are the potential implications of these results for disease biology and the possible impact on patients?**

It is important to generate more models of BMD in view of comparing them and identifying which dystrophin isoforms (and internal deletions) are associated with which phenotype. The latter is important for at least two reasons: (1) to provide better predictions about disease severity and phenotypes to patients and their families, and (2) to better understand the effects of treatments for different BMD variants. Additionally, our unbiased transcriptomic data now also offer insights into the pathophysiology of BMD and provides new directions for research (e.g. investigating the role of BMP signaling and the Gm family of long non-coding RNAs and their contribution to BMD pathology).

**Figure:**
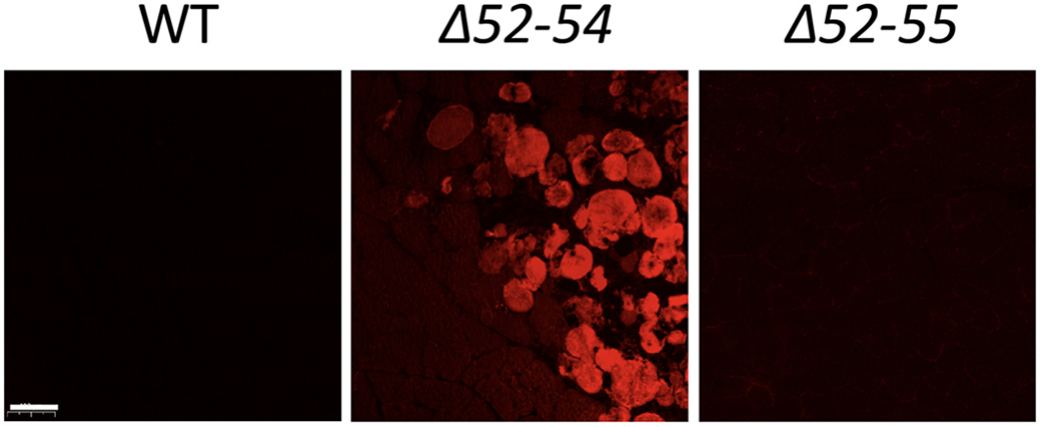
**Uptake of Evan's Blue dye in wildtype (WT) mice, mice with Duchenne muscular dystrophy (*Dmd* Δ52-54) and mice with Becker muscular dystrophy (*Dmd* Δ52-55).** Uptake of Evan's Blue dye is a way to visualize damaged muscle fibers. What is striking about this image is that 12-week-old mice with Becker's muscular dystrophy (right) show no more damaged fibers than WT mice (left), even though they have a multi-exonic deletion of *Dmd* exons 52 to 55. The differences in presentation and phenotype between patients (or models) with different variants in the *DMD* gene is fascinating to me. Scale bar: 100 μm.

**Why did you choose DMM for your paper?**

Disease Models & Mechanisms seemed to be an ideal journal for our paper given that it introduces a novel disease model and offers new insights into the pathophysiology of BMD. Given our lab's prior publication in DMM and the close connection between our previous and current research, we believed that DMM was the most suitable publication outlet. Additionally, DMM's commitment to promoting open science aligns with my own values and preferences for our field.

**Given your current role, what challenges do you face and what changes could improve the professional lives of other scientists in this role?**

One of the challenges my peers and I are facing is learning about the wide range of career options available to people who have completed a PhD in my field. Our university introduced a novel program aimed at giving us an overview of different career choices and enabling discussions with professionals who can tell us about their experiences. I wish these kinds of programs were available more broadly (maybe earlier in our career) and extended so that we are aware of all the choices available to us. In the same vein, I think creating collaboration between PhD students of different fields (for example, with students from bioethics, philosophy, engineering, etc.) would be very valuable.

**What's next for you?**

I am entering my fourth year of my PhD, and my current goal is to complete my research project, which is looking at developing and evaluating a therapeutic platform for individualized CRISPR/Cas9-based therapies for patients with Duchenne muscular dystrophy duplications. I am also working with the SickKids' Bioethics and Precision Child Health departments on identifying and addressing ethical issues around the administration of individualized therapies/*n*-of-1 trials. I would like to incorporate both aspects of my work in my future career.

**Tell us something interesting about yourself that wouldn't be on your CV**

Outside the lab, I enjoy doing photography. My passion for photography started after a trip to Cambodia in 2017 and has taken me to different workshops around the world, including Iceland, the UK and places closer to home in Ontario, Canada. I am also passionate about travelling and discovering new cultures in general and have had the chance to travel to over 35 countries. I have recently turned to black-and-white photography, which I really enjoy as it allows me to focus on interesting lines, shapes, patterns and light.
